# A New Genus and Species of Brachyscleromatinae (Hymenoptera: Ichneumonidae) from China, *Laxiareola ochracea*

**DOI:** 10.1673/031.011.0127

**Published:** 2011-03-08

**Authors:** Mao-Ling Sheng, Shu-Ping Sun

**Affiliations:** General Station of Forest Pest Management, State Forestry Administration, Shenyang, Liaoning, 110034, China

**Keywords:** Hymenoptera, Ichneumonidae, key to Brachyscleromatinae, *Laxiareola ochracea*

## Abstract

*Laxiareola* Sheng and Sun, gen.nov. and *Laxiareola ochracea* Sheng and Sun, sp.nov. belong to Brachyscleromatinae of the family Ichneumonidae, from the Jiangxi Province in China, are described in the present study. A key to the genera of Brachyscleromatinae is given.

## Introduction

Brachyscleromatinae, resurrected and diagnosis restated by Quicke et al. (2009), is a small subfamily belonging to family Ichneumonidae of Hymenoptera and comprises five genera including *Lygurus* Kasparyan 1983. Two genera, *Brachyscleroma* Cushman 1940 and *Lygurus* Kasparyan 1983, have been reported in China. In this article, one new genus and its type species collected in Quannan County, Jiangxi Province, China, are described. The type specimen is deposited in the Insect Museum, General Station of Forest Pest Management, State Forestry Administration, in the People's Republic of China.

The morphological terminology is mostly that of Gauld (1997). Wing vein nomenclature is based on Mason (1986, 1990).

## Description

***Laxiareola* Sheng and Sun, gen.nov.**
**Diagnosis.** Forewing about 8.6 mm long. Clypeal suture weak, not clearly separating face from clypeus. Clypeus almost flat, apical margin thick, with a fringe of parallel hairs. Mandible with two teeth, upper tooth longer than lower tooth. Antenna short; scape subcylindric, at least 2 times longer than its widest diameter; its apical truncation almost transverse. Occipital carina complete, middorsal portion horizontal. Notaulus weak, not reaching to center of mesoscutum. Upper end of epicnemial carina reaching to mid-height of hind margin of pronotum and distant from front margin of mesopleuron. Scutellum with lateral carina at basal 0.4. Areolet absent. Hind wing vein 1-cu strongly inclivous, at least 4 times as long as cu-a. Tarsal claw pectinate. Propodeum completely carinated. Area superomedia wider than long. First tergum strongly widened toward apex, approximately 1.8 times as long as its apical width, with deep glymmae. Second tergum with a longitudinal groove outside of the spiracle. Ovipositor sheath longer than hind tibia. Ovipositor (Figure 5) evenly upcurved, tip elongate, subapical portion of upper valve with nodus, lower valve with about 8 ridges, basal 4 widely spaced, distal 4 moderately close together.
**Type species.** *Laxiareola ochracea* Sheng and Sun, sp.nov.
**Distribution.** There is a single Chinese species, described below.
**Etymology.** The name of the new genus is based on very wide area superomedia, which is wider than it is long. The gender is female.

**Key to the genera of subfamily Brachyscleromatinae:**
1. Areolet closed by distinct or nebulous veins. Spiracle of first tergum behind mid-length. Glymma of first tergum absent. Apex of first sternite extending past middle of tergum. Apex of front tibia without a small tooth. *Brachyschleroma* Cushman
Areolet open. Spiracle of first tergum anterior to or at mid-length. Glymma of first tergum present. Apex of first sternite not extending past middle of tergum. Apex of front tibia with a small tooth. 2
2. Tarsal claws pectinate. Epomia present. Second tergum with a longitudinal groove outside of the spiracle Laxiareola Sheng and Sun, gen.nov.
Tarsal claws simple. Epomia absent. Second tergum without a longitudinal groove outside of spiracle, or with a longitudinal groove mesad of the spiracle. 3
3. Anterior transverse carina of propodeum absent. First tergum 1.0 to 1.5 as long as wide. *Melanodolius* Saussure
Anterior transverse carina of propodeum present. First tergum 2.5 to 5.0 as long as wide. 4
4. Clypeus centrally with weak transverse ridge. Second tergum without a longitudinal groove mesad of the spiracle. Ovipositor slender, apical third strongly upcurved and somewhat depressed *Lygurus* Kasparyan
Clypeus without transverse ridge. Second tergum with or without a longitudinal groove mesad of the spiracle. Ovipositor relatively stout or slende, apical portion not upcurved and weakly compressed or cylindric. 5
5. Clypeus rather flat, its apical margin with a median tooth (*Erythrodolius formosus* Seyrig 1932). Propodeum areolated. Second tergum with a longitudinal groove mesad of the spiracle. Ovipositor moderately thick, apical portion weakly compressed. *Erythrodolius* Seyrig
Clypeus weakly convex, its apical margin with a raw of tubercles. Propodeum only with anterior transverse carina and area basalis. Second tergum without a longitudinal groove mesad of the spiracle. Ovipositor rather slender, apical portion cylindric. *Icariomimus* Seyrig

**Figure 1.**
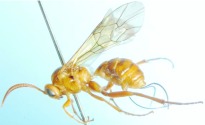
*Laxiareola ochracea* Sheng and Sun, gen.sp.nov. Body, lateral view. High quality figures are available online.

**Figure 2.**
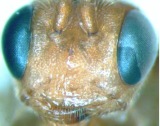
*Laxiareola ochracea,* Face. High quality figures are available online.

**Figure 3.**
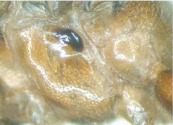
*Laxiareola ochracea,* Mesopleuron. High quality figures are available online.

**Figure 4.**
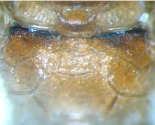
*Laxiareola ochracea,* Propodeum. High quality figures are available online.

**Figure 5.**
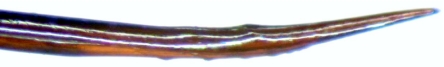
*Laxiareola ochracea,* Ovipositor. High quality figures are available online.

***Laxiareola ochracea* Sheng and Sun, sp.nov.  (Figures 1, 2, 3, 4, 5)**
Diagnosis
  Body yellowish brown. Speculum dark brown, smooth and shining. Antenna less than 0.7 length of forewing. Postero-ocellar line about 0.3 times as long as ocular-ocellar line. Hind wing vein 1-cu strongly inclivous, about 4.6 times as long as cu-a. Apical edge of first trochanter of leg with a small tooth on the outer side. Area superomedia very wide, approximately 1.8 times as wide as long. Ovipositor evenly upcurved.
Description
  Female. Body length about 9.3 mm. Forewing length about 8.6 mm. Antenna length about 5.5 mm. Ovipositor sheath length about 3.5 mm.
**Head.** Face (Figure 2) 2.0 times as wide as long, with dense punctures; median portion convex and smooth; upper median portion with a longitudinal protuberance. Clypeal suture indistinct. Clypeus almost flat, with unclear punctures; apical margin with a fringe of long parallel hairs, and a row of tubercles on median section. Mandible long, basal width nearly as wide as apex, its median portion slightly narrow; with shallow transverse punctures; upper tooth slightly longer than lower tooth. Malar space slightly rough, with unclear longitudinal lines, 0.6 times as long as basal width of mandible. Subocular sulcus indistinct. Gena nearly smooth, with sparse and fine punctures, in lateral view about 0.9 times as long as width of eye. Vertex with dense punctures, and deep concave nearby lateral ocellus. Interocellar area with punctures denser and finer than vertex. Postero-ocellar line about 0.3 times as long as ocular-ocellar line. Lower portion of frons concave, upper portion nearly the same texture as vertex. Antenna short, approximately 0.65 times as long as forewing. Scape almost cylindric, approximately 2.3 times as long as its widest diameter; apical truncation nearly transverse; with 24 flagellomeres, ratio of length from flagellomere 1 to 5 in proper order: 4.0:3.8:3.6:3.4:3.2. Occipital carina complete, middorsal portion approximately horizontal.
**Mesosoma.** Pronotum smooth, anterior portion narrowly with unclear fine punctures; lateral concave with short transverse lines; posterior portion with distinct fine punctures, more denser nearby upper margin. Epomia short, but distinct. Mesoscutum with dense punctures. Notaulus weak, as a vestige on front portion of mesoscutum. Scutellum convex, highest portion slightly behind center; almost smooth, with sparse and fine punctures; lateral carina reaching 0.4 its length. Postscutellum convex, strongly oblique forward. Mesopleuron (Figure 3) smooth, with sparse punctures. Speculum smooth and lucent, posterior margin slightly raised, and touching mesopleural suture. Around mesopleural fovea smooth and lucent. Sternaulus very weak, about half as long as mesopleuron. Metapleuron smooth, upper-anterior portion with fine and indistinct punctures. Submetapleural carina complete and strong. Wing gray-brownish hyaline, 1cu-a distad of 1-M, distance between them about 0.3 length of 1cu-a. Areolet absent. Vein 2rs-m basad of 2m-cu, distance between them about 0.7 length of 2rs-m. Vein 2-Cu 0.5 times as long as 2cu-a. Hind wing vein 1-cu strongly inclivous, about 4.6 times as long as cu-a. Apical edge of first trochanter of leg with a small tooth on the outer side. Apex of front tibia with a small tooth. Tarsal claws pectinate. Propodeum (Figure 4) completely areolate, dorsal profile (from base to posterior transverse carina) about 0.38 length of propodeum, posterior profile strongly sloping. Area basalis distinctly wider than long. Area superomedia approximately 1.8 times as wide as long, its lateral carinae weak. Area basalis and area superomedia with irregular wrinkles. Area externa with distinct punctures. Residual portion with indistinct fine punctures. Propodeal spiracle oval, slightly raised.
**Metasoma.** First tergum evenly and strongly narrowed toward base, well-proportioned convex, approximately 1.8 times as long as its apical width, with fine punctures; spiracle small, round, placed at midlength of the tergum, apex of sternite approximately at 0.2 of tergum. Glymmae very deep, separated from the grymma on opposite side only by a translucent partition. Second tergum approximately 0.6 times as long as its apical width, with fine and indistinct punctures; spiracle small, round, placed slightly in front of midlength of the tergum. Third and the following terga with brown fluff and indistinct punctures. Ovipositor (Figure 5) evenly upcurved, tip elongate, subapical portion of upper valve with a weak nodus, lower valve with 8 weak ridges, basal 4 widely spaced, distal 4 moderately close together.
**Color** (Figure 1). Yellowish brown. Antennae darkish brown. Upper-posterior corner of pronotum, small fleck behind spiracle of first tergum, oblique strip on lateral portion of second tergum and submedian transverse bands of third to sixth terga puce. Anterior fleck of middle lobe and longitudinal bands of lateral lobes of mesoscutum brownish black. Speculum shining blackish brown. Hind leg mostly reddish brown, its tarsi darkish brown.
**Type material**
  Holotype ♀, CHINA: Quannan, Jiangxi Province, 628 m, 12 May 2008, Mao-Ling Sheng.
**Distribution**
  China (Jiangxi)
**Etymology.** The name of the new species is based on the ochraceous color of body.
**Remarks.**
  The new genus resembles *Lygurus* Kasparyan 1983, but can be distinguished from the latter by the following characters: clypeus almost flat (without median transverse ridge); tarsal claw pectinate; first tergum strongly widened toward apex, approximately 1.8 times as long as its apical width; second tergum with a longitudinal groove outside of the spiracle; ovipositor sheath more shorter than body, less than 0.4 length of body; ovipositor comparatively strong, upper valve with nodus, lower valve with distinct ridges. *Lygurus* Kasparyan: clypeus with median transverse ridge; tarsal claw simple; first tergum strongly elongate, at least 3 times as long as its apical width; basolateral of second tergum with short groove; ovipositor sheath very long, 1.2 times as long as body; ovipositor slender, without nodus and ridge.
